# Effects of acute exercise training on tumor outcomes in men with localized prostate cancer: A randomized controlled trial

**DOI:** 10.14814/phy2.15408

**Published:** 2022-10-05

**Authors:** Sissal Sigmundsdóttir Djurhuus, Tim Schauer, Casper Simonsen, Birgitte Grønkær Toft, Adina Ruth Deborah Jensen, Janine Terra Erler, Martin Andreas Røder, Pernille Hojman, Klaus Brasso, Jesper Frank Christensen

**Affiliations:** ^1^ Centre for Physical Activity Research Copenhagen University Hospital – Rigshospitalet Copenhagen Denmark; ^2^ Department of Pathology Copenhagen University Hospital – Rigshospitalet Copenhagen Denmark; ^3^ Biotech Research and Innovation Centre (BRIC) University of Copenhagen (UCPH) Copenhagen Denmark; ^4^ Department of Urology Copenhagen Prostate Cancer Center, Copenhagen University Hospital –Rigshospitalet Copenhagen Denmark; ^5^ Department of Clinical Medicine University of Copenhagen Copenhagen Denmark; ^6^ The Department of Sports Science and Clinical Biomechanics Faculty of Health Sciences at the University of Southern Denmark Denmark; ^7^ Digestive Disease Center Bispebjerg Hospital Copenhagen Denmark

**Keywords:** acute exercise, cancer, high‐intensity exercise, immune cells, natural killer cells, prostate cancer, tumor hypoxia

## Abstract

Postdiagnosis physical activity is associated with improved cancer outcomes, but biological mechanisms mediating anticancer effects remain unclear. Recent findings suggest that physiological adaptations to acute exercise comprise potential anticancer effects, but these remain poorly explored in clinical settings. The objective of this study was to explore the effects of a single exercise bout on tumor oxygenation and immune cell infiltration in patients with prostate cancer. Thirty patients with localized prostate cancer were randomized (2:1) to either one high‐intensity interval training bout or no exercise on the day before radical prostatectomy. Immunohistochemical analyses were performed on prostatic tissue from surgery and assessed for tumor hypoxia, natural killer (NK) cell infiltration, and microvessel density (MVD). Acute systemic response in blood lymphocytes, epinephrine, norepinephrine, IL‐6, tumor necrosis factor, cortisol, lactate, and glucose was also evaluated. We did not find between‐group differences in tumor hypoxia (Mann–Whitney *U* test*, U* = 83.5, *p* = 0.604) or NK cell infiltration (*U* = 77.0, *p* = 0.328). Also, no significant correlation was found between MVD and tumor hypoxia or NK cell infiltration. One exercise bout is likely insufficient to modulate tumor hypoxia or NK cell infiltration. Future studies may elucidate if an accumulation of several exercise bouts can impact these outcomes (NCT03675529, www.clinicaltrials.gov).

## Graphical abstract

Biological mechanisms mediating anti‐cancer effects of physical exercise in humans are unclear, but physiological adaptations to acute exercise have been shown to hold anti‐cancer potential in preclinical studies. We explored the effects of one exercise bout on tumor oxygenation and immune cell infiltration in patients with prostate cancer and report that one exercise bout is not enough to impact these outcomes in a human setting, and the accumulation of several exercise bouts may be required to impact tumor biology.

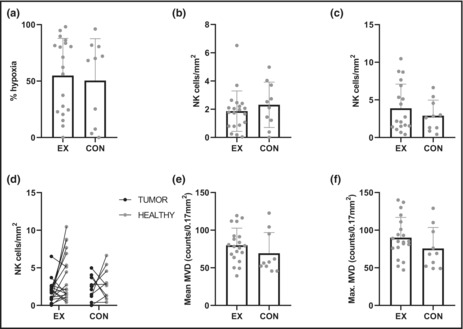

## 1 INTRODUCTION

Physical exercise training has been associated with improved outcomes after a cancer diagnosis, as observational studies demonstrate that high physical activity levels are associated with a lower risk of cancer recurrence and improved survival (Ballard‐Barbash et al., 2012; Kenfield et al., 2011; Richman et al., 2011). However, the underlying biological mechanisms responsible for the potential prognostic improvements are unclear, limiting the actionability of exercise training prescriptions as an integrated part of anticancer treatment.

To date, most clinical research in this space has focused on increasing physical activity levels with specific importance dedicated to the (long‐term) metabolic improvements associated with weight loss and the possible derived benefits of lowering systemic risk factors such as insulin, IGF‐1, and inflammatory markers. However, emerging preclinical evidence demonstrates that the transient physiological adaptations to a single exercise bout inhibit tumor cell viability (Aoi et al., 2013; Dethlefsen et al., 2016; Gannon et al., 2015; Hojman et al., 2011), suggesting that accumulation of repeated bouts of exercise, independently of long‐term physiological changes, holds the potential to induce anticancer effects.

Accordingly, seminal work in preclinical models has proposed different plausible mechanisms through which the accumulation of acute exercise bout may inhibit tumor growth, for example, effects on tumor physiology and immunogeniety (Dethlefsen et al., 2017; McCullough et al., 2013; Pedersen et al., 2016). Research from our group demonstrated that voluntary wheel running led to mobilization and redistribution of epinephrine‐sensitive natural killer (NK) cells to the tumors, partly by an interleukin (IL)‐6‐dependent mechanism leading to a suppression of tumor growth in mice (Pedersen et al., 2016). Also, voluntary or forced exercise has been shown to alter tumor vascularization and reduce tumor hypoxia in preclinical models (Betof et al., 2015; Garcia et al., 2016; McCullough et al., 2013; McCullough et al., 2014). These proposed mechanisms are supposedly driven by an accumulation of the marked acute effects: that is, exercise‐induced increase in systemic immune cell concentration and function, blood flow redistribution, and formation of new microvessels, which in concert may lead to favorable tumoral changes (Schumacher et al., 2021; Wiggins et al., 2018). However, the translation of these findings to human settings remains ambiguous, at least partly due to the challenge of distinguishing between acute signals and long‐term adaptations in human exercise interventions. Indeed, to our knowledge, no human studies to date have examined the tumor biological changes associated with a single acute exercise bout.

To this end, we investigated the effect of a single high‐intensity interval training (HIIT) bout in patients with localized prostate cancer (PCa) undergoing radical prostatectomy. This population does not receive neo‐adjuvating treatment and typically presents with slow‐growing tumors, making exercise‐conditioned tumor tissue obtainable without subjecting patients to additional tumor sampling or delays in treatment plans with implications for long‐term prognosis. Our primary objective was to examine whether a single bout of HIIT performed on the day before surgery leads to differences in intratumoral NK‐cell infiltration and/or hypoxia level compared with a nonexercise control setting. Second, we explored if these markers (NK cell and hypoxia levels) were associated with tumor microvessel density (MVD) as tumor perfusion may be a determining factor for exercise‐induced adaptations.

## 2 PATIENTS AND METHODS

This randomized controlled trial included 30 patients with histologically verified localized prostate adenocarcinoma scheduled for radical prostatectomy. The participants were recruited from the Urologic Department at Rigshospitalet, Copenhagen University Hospital, Denmark, from October 2018 to November 2019. Exclusion criteria were age <18 years, other malignancy requiring active treatment; Eastern Clinical Oncology Group (ECOG) performance status score > 1; current treatment with beta‐adrenergic receptor antagonists; physical disabilities contradicting exercise; allergy to pimonidazole, and the inability to read and understand Danish.

Patients were identified preoperatively by the urologic physicians, followed by a telephone screening for eligibility. Patients underwent baseline assessment on the day before surgery, comprising medical examination and anthropometrics. Following assessment, patients were randomized to either one acute aerobic HIIT bout group (EX) or a control (CON) group. Randomization was performed using a computer‐generated random allocation sequence and based on block randomization with a block size of three and an allocation ratio of 2:1. An online clinical trial software (easytrial.net) ensured allocation concealment, keeping the allocation sequence unavailable for the investigators and participants. All analyses were performed blinded to group allocation.

To assess tumor hypoxia, participants ingested pimonidazole hydrochloride capsules (Hypoxyprobe Inc.,) at a dosage of 500 mg/m^2^. Pimonidazole was administered in immediate continuation of the exercise bout in the EX group. In the CON group, pimonidazole was administered after the randomization. Patients were fasting 2 h before the baseline assessment.

The study was conducted at the Centre for Physical Activity Research, Rigshospitalet, Copenhagen University Hospital, Denmark and approved by the local Ethics Committee of the Capital Region of Denmark (H‐18020711). All participants provided informed consent before performing any study‐related procedures, and the study was preregistered at www.clinicaltrials.gov (NCT03675529).

### 2.1 Intervention

The exercise session first consisted of determining aerobic capacity by a peak power output (W_peak_) test followed by an aerobic HIIT bout. The W_peak_ test was performed on a bicycle ergometer (LC4, Monark Exercise AB) with 3 min of warm‐up at 70 W followed by an incremental increase of 20 W every minute until exhaustion. After a 10 min active recovery at 30% of W_peak_, participants performed four high‐intensity (HI) interval cycles on a stationary bicycle ergometer consisting of 1 min at 100% W_peak_, followed by 3 min of active recovery at 30% of W_peak_. The intensity of the HI intervals was determined from the W_peak_ test. We estimated VO_2_peak (ml O_2_/min) using the American College of Sports Medicine's 6th edition guidelines for leg cycling ergometry: 7 + 10.8 * W_peak_/weight (kg) (Swain, 2000). Rating of perceived exertion (RPE) was performed using the Borg (Aoi et al., 2013; Betof et al., 2015; Borg, 1982; Carnell et al., 2006; Dethlefsen et al., 2017; Gannon et al., 2015; Garcia et al., 2016; McCullough et al., 2013; McCullough et al., 2014; Pedersen et al., 2016; Ragnum et al., 2015; Raleigh et al., 2001; Schumacher et al., 2021; Swain, 2000; Wiggins et al., 2018) scale (Borg, 1982) and was obtained immediately after the W_peak_ and each HI interval.

### 2.2 Outcome measures

#### 2.2.1 Tumor hypoxia

Slides from formalin‐fixed, paraffin‐embedded (FFPE) prostatic tissue blocks from radical prostatectomy were used for all immunohistochemical (IHC) analyses. Tumor hypoxia, being the study's primary outcome, was assessed in three different tumor foci in the prostate. First, the tissue block containing the largest tumor area was selected. Second, two additional foci throughout the prostate were identified. Tumor hypoxia was assessed using the hypoxia marker pimonidazole hydrochloride, a bioreductive 2‐nitroimidazole compound that covalently forms protein adducts detectable by IHC in hypoxic tissue with a pO_2_ ≤ 10 mm Hg at 37°C (Carnell et al., 2006; Ragnum et al., 2015; Raleigh et al., 2001; Varia et al., 1998). Immunohistochemical staining was performed using the Hypoxyprobe 1 MAB1 Mouse IgG1 antibody (Hypoxyprobe Inc.,), following the manufacturer's instructions. Following initial incubation, de‐paraffinizing, and rehydration, sections were heated in antigen retrieval buffer. After cooling, slides were washed in dH_2_O, followed by incubation in 3% H_2_O_2_. Sections were washed in Tris‐buffered saline with tween (TBS‐T) buffer, drawn around each section with a waterproof pen, and blocked for 60 min. Next, sections were incubated overnight with the primary antibody at 4°C. On the second day, slides were placed in TBS‐T buffer, and DAKO HRP Mouse antibody (Agilent Technologies) was added. Next, slides were washed in TBS‐T buffer, and DAB Chromogen (Peroxidase) (Vector Laboratories) was added as per kit instructions. Sections were counterstained with Mayer's hematoxylin (Sigma‐Aldrich).

Stained slides were scanned using the Hamamatsu NanoZoomer‐XR (Hamamatsu) whole slide scanner with ×40 magnification. Hypoxia quantification was performed in ImageJ (Rueden et al., 2017). All images were imported, and the threshold was set for all. The total area of the tumor and hypoxia were measured manually, and the hypoxic fraction of the tumor (positive pimonidazole staining) was determined.

#### 2.2.2 NK cell infiltration

Tissue sampling for IHC analysis and NK cell infiltration assessment was conducted by first identifying the diagnostic core needle biopsy containing the highest adenocarcinoma infiltration (percentage of tumor tissue). Second, the corresponding area in the prostatectomy specimen was located, and the tumor focus was identified.

Immunohistochemistry was performed using standard methods. We immunostained tissue sections (3 μm thick) using the CD56 antibody (Roche Diagnostics International AG) following the manufacturer's instructions. We used PT Link (Agilent Technologies) with a high pH/low pH target retrieval solution (Dako DM828) to pretreat tissue sections. The staining took place using the Ready‐To‐Use (RTU) format on the DakoLink 48 (Agilent Technologies) utilizing the EnVision Flex+ detection kit (K8002). Tissue sections were incubated for 20 min and counterstained with hematoxylin.

We digitalized the stained slides using the Hamamatsu Nano Zoomer‐XR at a magnification equivalent to ×20 magnification and manually quantified the NK cells using the Hamamatsu viewing software NDP.View (version 2.6.13, Hamamatsu Photonics) at ×40 magnification. NK cell infiltration was determined as the number of NK cells (cells/mm^2^) in the entire tumor area. In the healthy prostatic tissue, NK cell quantification was performed in the same manner as for the tumor tissue but in a randomly chosen area of the same size as the tumor area.

#### 2.2.3 Microvessel density

Tumor MVD was assessed by IHC using standard methods. Tissue sections (3 μm) were stained using the CD31 antibody (Agilent Technologies) following the manufacturer's instructions. The staining was performed on the Omnis platform (Agilent Technologies) with the EnVision Flex+ detection kit (GV800) using the RTU format. Incubation time was 20 min, and sections were counterstained with hematoxylin.

Stained slides were scanned using the Hamamatsu Nano Zoomer‐XR at a magnification equivalent to ×20 magnification. Tumor MVD was assessed using the method introduced by Weidner (Kraby et al., 2019; Rogatsch et al., 1997; Weidner et al., 1993) and manually quantified using the Hamamatsu viewing software NDP.View (version 2.6.13, Hamamatsu Photonics). First, tumor sections were scanned at low magnification (×4 and ×10) to identify areas of high neovascularization (“hot spots”). Second, the quantification was performed in one visual field (0.17 mm^2^) from three randomly chosen neovascular hot spots at high (×40) magnification. The MVD was expressed as both the mean and the highest MVD from the three fields.

#### 2.2.4 Blood biochemistry

In the EX group, blood samples including total lymphocyte count, cortisol, interleukin (IL)‐6, tumor necrosis factor (TNF)‐α, lactate, and glucose were measured at four time points during the exercise bout: (Ballard‐Barbash et al., 2012) at baseline, (Kenfield et al., 2011) immediately after the W_peak_ test, (Richman et al., 2011) immediately after the last HI, and (Dethlefsen et al., 2016) 1 h into the resting period. High‐sensitivity (hs)‐CRP and Prostate‐Specific Antigen (PSA) were measured only at baseline. Total lymphocyte count, cortisol, and PSA were measured by standard laboratory analyses at the Department of Clinical Biochemistry, Rigshospitalet, Copenhagen University Hospital, Denmark. For the assessment of hs‐CRP, IL‐6, TNF, epinephrine, and norepinephrine, blood was collected in EDTA‐containing tubes, followed by plasma isolation via centrifugation (2000 g, 10 min, 4°C), short‐term storage at 4°C, and long‐term storage at −80°C within 2 h. Hs‐CRP, IL‐6, and TNF were assessed according to the manufacturer's instructions (Human CRP kit and pro‐inflammatory panel 1, Meso Scale Discovery). Lactate and glucose were assessed using the ABL800 FLEX PLUS (Radiometer Medical) blood gas analyzer. Epinephrine and norepinephrine were measured with the 2‐CAT ELISA Fast Track Elisa (LDN) according to the manufacturer's guidelines.

In the CON group, blood samples for analyses of total lymphocytes, cortisol, PSA, hs‐CRP, IL‐6, and TNF were collected in the morning before the surgery.

### 2.3 Statistical analyses

We aimed to include a minimum of 30 patients. No formal power calculations could be made due to the lack of studies reporting tumor hypoxia assessed by pimonidazole staining in humans. Analysis of tumor hypoxia, NK cell infiltration, and MVD was performed using the Mann–Whitney U test. To analyze the response in blood parameters during the exercise bout, we used a linear mixed model analysis with concentration as the outcome variable and time point as a fixed effect with baseline concentration as a covariate and a random effect of participants. Data were log‐transformed to improved model compliance and back‐transformed as relative ratios; thus, an estimate of 1.10 corresponds to a median relative change of 10%. For correlations analyses, we used Pearson's bivariate correlation or Spearman's rho correlation analyses depending on the normal distribution of the data. Raw baseline data were expressed as mean or median for all outcomes with corresponding standard deviation (SD) or interquartile range (IQR), respectively. Analyses of the prespecified outcomes were performed as intention‐to‐treat analyses. Analyses were performed using IBM SPSS Statistics for Windows, Version 25.0. The mixed model analysis was performed using R (The R Foundation for Statistical Computing, Vienna, Austria) and the lme4 package (Bates et al., 2015). A two‐tailed *p* < 0.050 is reported as statistically significant.

## 3 RESULTS

### 3.1 Participants

**FIGURE 1:**
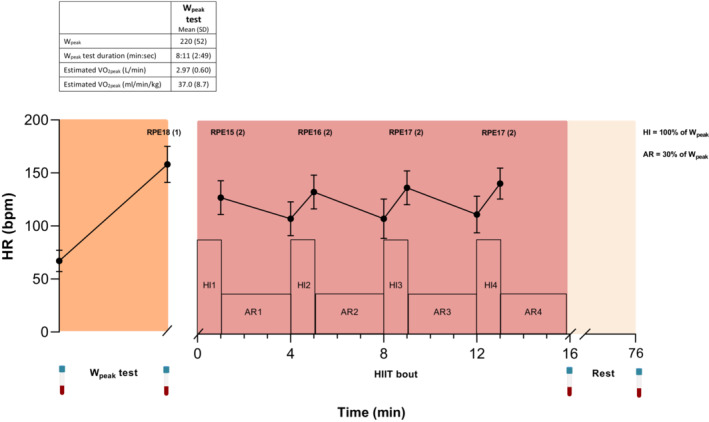
HR response and RPE (mean [SD]) during W_peak_ test and HIIT bout for all participants (*n* = 20) in the EX group. Blood samples (icon) were collected at baseline, immediately after W_peak_ test and HIIT bout, and after 1 h of rest. AR, active recovery, bpm, beats per minute; Ex, exercise; HI, high‐intensity interval; HIIT, High‐Intensity Interval Training; HR, heart rate, PRE, rate of perceived exertion; VO_2peak_, peak oxygen consumption; W_peak_, peak power output.

Seventy patients were screened for eligibility, and 30 were enrolled in the study and randomized (Table 1). In total, 20 out of 30 patients performed one bout of acute exercise on the day before the scheduled prostatectomy. All participants completed the HIIT bout with no reductions in prescribed exercise intensity. Physiological parameters during the exercise bout are presented in Figure 1. The mean W_peak_ was 220 (SD 52) with a mean W_peak_ test duration of 8:11 (2:49) min. The estimated VO_2peak_ was 37.0 (8.7) ml/min/kg.

**TABLE 1:** Participants characteristics

	CON group (*n* = 10)	EX group (*n* = 20)
Age (years), mean (SD)	65 (6)	64 (6)
Gleason score, *n* (%)		
<7	1 (10%)	5 (25%)
7	5 (50%)	11 (55%)
>7	4 (40%)	4 (20%)
PSA (μg/L), median (Q1; Q3)	12.5 (7.1; 23.8)	7.9 (6.6; 11.8)
BMI (kg/m^2^), mean (SD)	25.3 (2.9)	26.3 (3.4)
Weight (kg), mean (SD)	78.8 (10.6)	81.7 (12.1)
Waist circumference (cm), mean (SD)	99.7 (10.0)	99.1 (9.8)
Waist‐to‐height ratio, mean (SD)	0.57 (0.05)	0.56 (0.06)
Blood pressure (mm Hg), mean (SD)		
Systolic	141 (15)	139 (13)
Diastolic	90 (9)	85 (10)
Resting HR (bpm), mean (SD)	68 (17)	67 (10)
Blood parameters, median (Q1; Q3)		
Lymphocytes (10^9^/L)	1.80 (1.48; 1.93)	1.70 (1.40 1.90)
Cortisol (nmol/L)	369 (238; 471)	214 (157; 280)
Hs‐CRP (mg/L),	0.52 (0.22; 1.17)	2.33 (0.49; 4.09)
IL‐6 (pg/ml),	0.43 (0.35; 0.83)	0.64 (0.51; 0.98)
TNF (pg/ml)	2.32 (1.87; 3.00)	2.55 (2.06; 3.67)
Smoker, *n* (%)		
Never	6 (60.0%)	8 (42.1%)
Previous	4 (40.0%)	10 (52.6%)
Current	0 (0.0%)	1 (5.3%)
Alcohol intake (units/week), *n* (%)		
≤14	10 (100.0%)	12 (63.2%)
>14	0 (0.0%)	7 (36.8%)
Physical activity level (min MVPA/week), *n* (%)		
<150 min	4 (40%)	4 (20.0%)
≥150 min	6 (60.0%)	16 (80.0%)
NK‐cell infiltration (diagnostic biopsy), median (Q1; Q3)		
NK cells/mm^2^ tumor tissue	1.04 (0.19; 2.86)	1.33 (0.27; 2.49)
NK cells/mm^2^ healthy tissue	3,80 (0.94; 10.37)	3.57 (1.21; 6.10)

*Note*: Values are presented as mean (SD), median (Q1 and Q3), or *n* (%).

Abbreviations: BMI, body mass index; CON, control; EX, exercise; HR, heart rate; hs, high sensitivity; IL, interleukin; MVPA, moderate‐to‐vigorous physical activity; NK, natural killer; PSA, Prostate‐Specific Antigen; TNF, tumor necrosis factor.

### 3.2 Systemic physiological response

**FIGURE 2:**
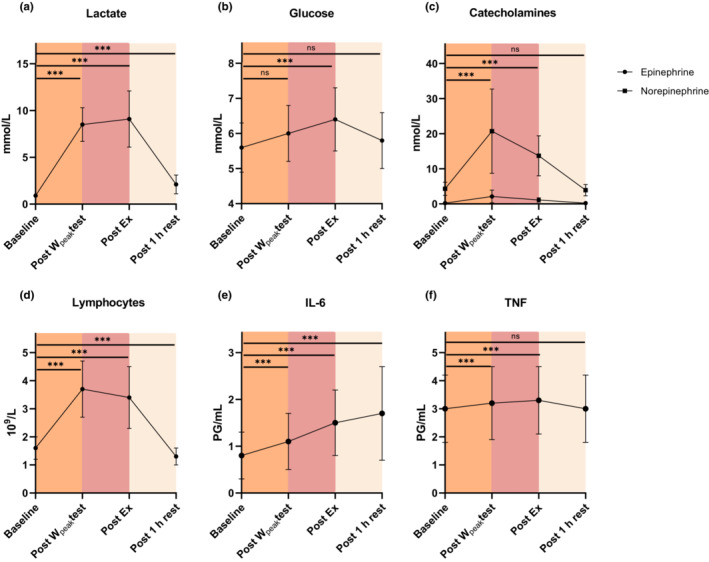
Acute systemic response in (a) lactate, (b) glucose, (c) catecholamines, (d) lymphocytes, (e) IL‐6 and (f) TNF before, during, and after the EX bout. Abbreviations: Ex, exercise; IL, interleukin; TNF, tumor necrosis factor; W_peak_, peak power output.

Blood parameters before, during, and after the exercise bout are presented in Figure 2 and Table 2. We observed an increase in catecholamines, cortisol, lactate, and glucose immediately post exercise. In addition, the total blood lymphocyte count increased during the exercise bout, with the highest response during the W_peak_ test, and with a significant decrease below baseline values after 1 h of rest. We observed a significant increase in IL‐6 throughout the exercise bout, with the highest levels observed 1 h postexercise. Last, TNF increased to a lesser extent during the exercise bout.

**TABLE 2:** Systemic physiological response during acute exercise

	Raw data mean (SD)				Change from baseline change (95% CI), *P* value		
	Baseline	Post‐W_peak_ test	Postexercise	Post‐1 h rest	Post‐W_peak_ test *P*	Postexercise *P*	Post‐1 h rest *P*
Lactate (mmol/L)	0.9 (0.2)	8.5 (1.8)	9.1 (3.0)	2.1 (1.0)	9.77 (8.11; 11.77) <0.001	10.13 (8.41; 12.21) <0.001	2.32 (1.91; 2.83) <0.001
Glucose (mmol/L)	5.6 (0.7)	6.0 (0.8)	6.4 (0.9)	5.8 (0.8)	1.06 (1.0; 1.13) 0.052	1.14 (1.07; 1.21) <0.001	1.04 (0.97; 1.10) 0.481
Lymphocytes (10^9^/L)	1.6 (0.4)	3.7 (1.0)	3.4 (1.1)	1.3 (0.3)	2.23 (2.0; 2.5) <0.001	2.01 (1.79; 2.24) <0.001	0.79 (0.70; 0.88) <0.001
Epinephrine (nmol/L)	0.2 (0.2)	2.1 (1.8)	1.1 (0.6)	0.2 (0.1)	8.01 (5.13; 12.51) <0.001	4.75 (3.04; 7.41) <0.001	0.80 (0.51; 1.25) 0.541
Norepinephrine (nmol/L)	4.3 (1.9)	20.7 (12.0)	13.7 (5.7)	3.9 (1.6)	4.50 (3.52; 5.75) <0.001	3.24 (2.54; 4.15) <0.001	0.91 (0.71; 1.16) 0.697
Cortisol (nmol/L)	216.9 (71.0)	238.7 (103.9)	527.4 (173.9)	472.3 (180.8)	0.97 (0.63; 1.49) 0.997	2.32 (1.50; 3.59) <0.001	2.11 (1.37; 3.25) <0.001
IL‐6 (pg/ml)	0.8 (0.5)	1.1 (0.6)	1.5 (0.7)	1.7 (1.0)	1.42 (1.18; 1.70) <0.001	1.86 (1.55; 2.23) <0.001	2.00 (1.66; 2.40) <0.001
TNF (pg/ml)	3.0 (1.2)	3.2 (1.3)	3.3 (1.2)	3.0 (1.2)	1.09 (1.05; 1.14) <0.001	1.10 (1.06; 1.15) <0.001	1.00 (0.96; 1.05) 0.994

*Note*: Means (SD) are based on all available data for the EX group at four time points throughout the exercise bout. n = 20 for all time points, except for cortisol postexercise *n* = 19. The mean changes are estimated means from the linear mixed models, and therefore, the changes may not reflect the numerical changes between each time point. Data are analyzed on log‐transformed data, and change estimates are back‐transformed and reported as median relative changes with back‐transformed 95% CIs.

Abbreviations: CI, 95% confidence interval; EX, exercise; IL, interleukin; PSA, prostate‐specific antigen; TNF, tumor necrosis factor; W_peak_, peak power output.

### 3.3 Tumor effects

**FIGURE 3:**
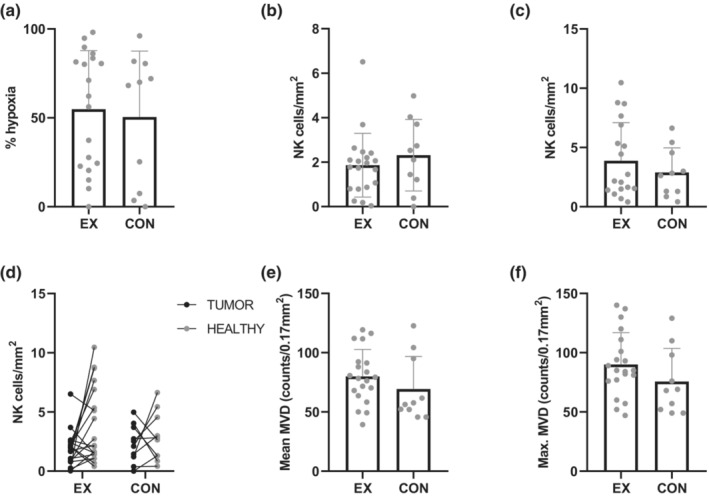
Data for (a) tumor hypoxia and NK cell infiltration in (b) tumor, (c) healthy prostatic tissue, and (d) the individual data points for NK cell infiltration in tumor and healthy tissue for each participant in the EX and CON groups. (e) The mean and (f) the highest MVD count in tumors in the EX and CON groups. All data except (d) are presented as mean bars with individual data points. CON, control; EX, exercise; MVD, microvessel density; NK, natural killer.

Data on tumor effects are presented in Figure 3 and Table 3. There was no difference in overall tumor hypoxia between the EX and CON groups (Mann–Whitney U Test*, U* = 83.5, *p* = 0.604) (Figure 3a). The mean hypoxic fraction of the tumor was 54.8% (SD 33.0) and 50.5% (37.1)% in the EX and CON groups, respectively. In two participants (EX = 1 and CON = 1), the tumors were nonhypoxic with 0% positive pimonidazole staining. One participant in the EX group had only one tumor focus and was therefore excluded from the hypoxia analysis.

**TABLE 3:** Tumor effects

	Raw data mean (SD)	*n*	Mean rank	*U* value	*p* value
Tumor hypoxia (percentage, %)					
EX	54.8 (33.0)	19	15.61	83.5	0.604
CON	50.5 (37.1)	10	13.85		
MVD, mean of three foci (counts/0.17 mm^2^)					
EX	80.0 (22.7)	20	17	70	0.198
CON	69.3 (27.5)	10	12.5		
MVD, max. of three foci (counts/0.17 mm^2^)					
EX	90.1 (26.8)	20	17.27	64.5	0.12
CON	75.7 (27.8)	10	11.95		
NK cell infiltration, tumor (cells/mm^2^)				77	0.328
EX	1.86 (1.43)	20	14.35		
CON	2.31 (1.61)	10	17.8		
NK cell infiltration, healthy (cells/mm^2^)				89	0.65
EX	3.79 (3.15)	20	16.05		
CON	2.90 (2.07)	10	14.04		
NK cell infiltration, tumor (total cells)				88.5	0.619
EX	169.6	20	16.08		
CON	135.1	10	14.35		
NK cell infiltration, healthy (total cells)				97	0.914
EX	222.45	20	15.65		
CON	179.4	10	15.2		

*Note*: Results from the Mann–Whitney *U* test for tumor outcomes in the EX and CON group.

Abbreviations: CON, control; EX, exercise; MVD, microvessel density; NK, natural killer.

In the EX and CON groups, the mean tumoral NK cell infiltration was 1.86 (SD 1.43) and 2.31 (1.61) cells/mm^2^, respectively. No between‐group difference in tumor NK cell infiltration was observed (Mann–Whitney U test, *U* = 77.0; *p* = 0.328) (Figure 3b). Nor was there any between‐group difference in NK cells in healthy prostatic tissue (Mann–Whitney U test, *U* = 89.0; *p* = 0.650) (Figure 3c). The mean NK cell infiltration in healthy tissue was 3.79 (3.15) and 2.90 (2.07) cells/mm^2^ in the EX and CON groups, respectively. Figure 3d shows the individual data points for NK cell infiltration in tumor and healthy tissue in the EX and CON groups. When assessing the total NK cell count without adjusting for tumor area (mm^2^), we did not observe any between‐group differences in NK cell infiltration in tumor (Mann–Whitney U test, *U* = 88.5; *p* = 0.619) or healthy tissue (Mann–Whitney U test, *U* = 97.0; *p* = 0.914) (Table 3).

MVD data are presented in Figure 3e,f and Table 3. Mean MVD from the average of the three foci was 80.0 (SD 22.7) and 69.3 (27.5) counts/0.17 mm^2^ in the EX and CON groups, respectively. The mean MVD from the focus containing the highest MVD was 90.1 (26.8) and 75.7 (27.8) counts/0.17 mm^2^, respectively. Neither the mean MVD from the three foci (Mann–Whitney U Test, *U* = 70.0; *p* = 0.20) nor the foci with the highest counts/0.17mm^2^ field (Mann–Whitney U Test, *U* = 64.5; *p* = 0.12) differed between the EX and CON groups.

### 3.4 Exploratory analyses

**FIGURE 4:**
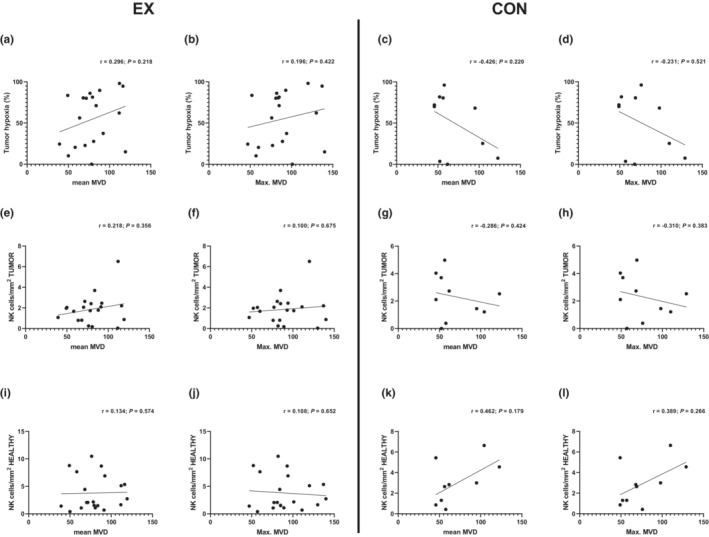
Exploratory analyses showing correlations between MVD and (a–d) tumor hypoxia, (e–h) tumor NK cell infiltration, and (i–l) NK cell infiltration in healthy prostatic tissue in the EX and CON groups. CON, control; EX, exercise; MVD, microvessel density; NK, natural killer.

As tumor vascularity plays a pivotal role in immune cell delivery and tumor hypoxia development, we aimed to explore the correlation between vascularity, assessed as MVD, and tumor hypoxia and NK cell infiltration. No significant correlation between MVD and tumor hypoxia or NK cell infiltration in tumor or healthy tissue was found in either the EX or CON group (Figure 4a–i).

## 4 DISCUSSION

Exercise training has been demonstrated to improve cancer outcomes in observational studies, and preclinical studies have shown that exercise may directly influence tumor biology (Ballard‐Barbash et al., 2012; McCullough et al., 2013; McCullough et al., 2014; Pedersen et al., 2016). However, the translatability of these mechanisms to a human setting is uncertain. Therefore, we designed the present study to explore and characterize the acute effects of one exercise bout on tumor hypoxia and NK cell infiltration. Here, one acute HIIT bout did not result in changes in tumor hypoxia or NK cell infiltration, suggesting that a single exercise session in this setting is insufficient to alter tumoral outcomes.

Studies have demonstrated that long‐term exercise can alter tumor vascularization and lower hypoxia in preclinical settings, potentially due to increased blood flow during exercise (Betof et al., 2015; McCullough et al., 2013; Schumacher et al., 2021), as first proposed by McCollough et al. in a mouse model of orthotopic prostate cancer (McCullough et al., 2014). Additionally, an increase in the number of open tumor vessels and a blunted vasoconstriction of tumor arterioles in response to norepinephrine was shown, reflecting the vasoconstrictive inability of the tumor vessels. These data suggest that exercise may hold the potential to increase tumor oxygenation by increasing perfusion and reducing hypoxia. To our knowledge, this hypothesis has only been explored in two clinical studies, with diverse results. Jones et al. explored the effect of aerobic exercise on markers of tumor hypoxia, MVD, and tumor blood flow in women with breast cancer receiving neoadjuvant chemotherapy and found no differences in MVD or markers of hypoxia but reported a decrease in tumor blood flow in the exercise group (Jones et al., 2013). However, in the study, pre–post tissue samples and PET scans were only available in a subset of patients. Contrarily, Florez Bedoya et al. reported that preoperative home‐based aerobic and resistance exercise in pancreatic cancer patients treated with chemo‐ or chemoradiation therapy led to remodeled tumor vasculature with increased MVD and more normalized vessels (Florez Bedoya et al., 2019).

During exercise, there is an increase in cardiac output and mean arterial pressure (MAP). Meanwhile, the newly formed tumor vessels are immature and thus lack smooth muscle cells, making them unable to respond to the exercise‐induced increase in norepinephrine and subsequent vasoconstriction (Jain, 1988; Wiggins et al., 2018). As vessel diameter is the dominant parameter in flow regulation (Hojman et al., 2018), it could be speculated that the increased systemic blood flow and MAP during exercise, together with the reduced myogenic constriction of the tumor vessels in response to norepinephrine, may increase tumor vessel diameter resulting in enhanced tumoral blood flow (Wiggins et al., 2018). Additionally, studies suggest that the potential increase in tumoral blood flow may induce an augmented immune cell delivery to the tumor (Hojman et al., 2018; Schumacher et al., 2021; Wiggins et al., 2018). However, in our study, the relatively short exercise duration (W_peak_ test followed by 4 × 1 min HI) may be too short to elicit a response in the tumor immune cells or hypoxia, that is, that tumor blood flow was not increased for a long enough period. Last, tumor vessels display decreased intravascular pressure and increased interstitial pressure leading to vessel collapse and reduced blood flow (Jain, 1988; Wiggins et al., 2018), which could have contributed to our results.

Assuming that exercise can induce alterations in tumor blood flow, it is unknown whether this would translate into a reduction of tumor hypoxia. In fact, tumor vasculature is very heterogeneous, immature, and characterized by tortuous, leaky vessels that are unequally distributed, resulting in uneven tumor blood flow (Carmeliet & Jain, 2000; Nagy et al., 2009). In continuation, anastomoses within the tumor shunt the oxygenated blood away from the tumor microcirculation where the gas exchange occurs, leading to reduced oxygen delivery and acute perfusion‐related hypoxia (Vaupel & Harrison, 2004). Supposing that this also occurs during exercise, it would increase tumor blood flow, but without subsequent reduction in tumor hypoxia.

Tumor immune cell infiltration is associated with improved cancer outcomes and constitutes another possible (synergetic) mechanism through which exercise can directly impact tumor growth (Cózar et al., 2021; Hojman et al., 2018; Melaiu et al., 2020; Pasero et al., 2016). Specifically, in a preclinical model, we have previously shown that exercise mobilizes epinephrine‐sensitive NK cells to the circulation, which then infiltrate tumors by an IL‐6‐dependent mechanism leading to a reduction in tumor growth (Pedersen et al., 2016). The increased NK infiltration was observed in response to a few weeks of exercise; however, it is well described that during acute exercise, there is a surge in the number of circulating immune cells due to adrenergic signaling and blood flow‐induced shear stress on the vessels (Idorn & Hojman, 2016). The mobilized immune cells engage in surveillance to identify potential transposed cells, and after cessation of exercise, the number of mobilized immune cells in the circulation falls below baseline levels (Hanson et al., 2020; Idorn & Hojman, 2016; Pedersen & Hoffman‐Goetz, 2000). The dip in immune cells to below preexercise levels may indicate homing to the tumor and subsequent tumoral immune cell infiltration (Pedersen & Hoffman‐Goetz, 2000). However, our data do not indicate tumoral changes in NK cell infiltration after one acute bout of exercise.

As expected with high‐intensity exercise, plasma epinephrine levels increased during the exercise bout, with the highest level after the W_peak_ test. In concert, it is well established that NK cells are mobilized to the circulation during exercise in an epinephrine‐dependent manner (Idorn & Hojman, 2016; Kappel et al., 1991), and indeed, we did see a substantial increase in the number of mobilized cytotoxic NK cells in the circulation during the exercise bout (data in submission). Thus, it was not due to an insufficient epinephrine response or lack of an NK cell mobilization that we did not demonstrate an increased tumor NK cell infiltration.

The present study has important limitations. We did not collect preintervention tumor samples for hypoxia assessment; thus, we could not adjust for baseline tumor hypoxia levels. Also, while PCa patients undergoing radical prostatectomy serve as an enticing study cohort, investigating the effects of exercise on tumor hypoxia and NK cell infiltration in this cohort is challenging. Notably, PCa is a multifocal and heterogeneous disease, and accordingly, three tumor foci throughout the prostate may not be sufficient for a valid analysis. Also, CD56 is not NK cell‐specific but is also expressed on CD8 and γδ T cells (Van Acker et al., 2017). Nonetheless, most if not all cells harboring CD56 are associated with exercise‐induced regulation of tumor growth.

Another critical challenge in investigating the acute effects of exercise on tumor outcomes is the current lack of knowledge of what exercise mode could elicit these potential changes in the tumor microenvironment. While the exploration of single exercise bouts is needed to advance this knowledge, it is likely that any significant impact on tumor biology requires the accumulation of repeated exercise bouts. To this end, we have recent data (in submission) indicating a dose–response relationship between the number of exercise sessions and tumor NK‐cell infiltration, supporting that one bout is not sufficient. The present mechanisms proposedly require longer interventions to induce measurable tumor effects. Another important consideration for interpreting the present finding is the timing of the pimonidazole administration in relation to the exercise bout. We administered pimonidazole orally immediately after the exercise bout. Given that the peak plasma concentration for oral pimonidazole is achieved after 2.5 h, combined with the unknown duration of the potential exercise effect on the tumor vasculature and perfusion, we could have missed a window of opportunity to measure a reduction in tumor hypoxia. Accordingly, future studies are warranted to investigate the effect of preexercise administration.

In summary, one acute HIIT bout on the day before surgery in localized PCa patients undergoing radical prostatectomy did not result in differences in hypoxia or NK cell infiltration compared with nonexercising controls. Further, we did not find an association between these tumor outcomes and MVD. One exercise bout might not be enough to modulate tumor hypoxia or NK cell infiltration, and an accumulation of single exercise bouts may be required to impact these outcomes.

## AUTHORS' CONTRIBUTION

All study‐related experiments were conducted at the Centre for Physical Activity Research, Rigshospitalet, Copenhagen University Hospital, Denmark. SSD conceptualized and designed the work, conducted the experiments, analyzed and interpreted the data, and wrote the manuscript. JFC, conceptualized and designed the work, interpreted the data, and wrote the manuscript. PH conceptualized and designed the work. BGT conceptualized, designed, and critically revised the work. KB conceptualized and designed the work, recruited patients, and critically revised the work. TS conducted the experiments, analyzed the data, and wrote the manuscript. CS analyzed and interpreted the data and critically revised the work. ARDJ and JTE analyzed the data and wrote the manuscript. MAR recruited patients and wrote the manuscript. All the authors approved the final version of the manuscript and agreed to be accountable for all aspects of the work in ensuring that questions related to the accuracy or integrity of any part of the work are appropriately investigated and resolved. All persons designated as authors qualify for authorship, and all those who qualify for authorship are listed.

## FUNDING INFORMATION

The study was funded by the TrygFonden and the Lundbeck Foundation.

## CONFLICT OF INTEREST

The authors declare that they have no conflict of interest.
